# The complete mitochondrial genome of the Bechstein’s bat, *Myotis bechsteinii* (Chiroptera, Vespertilionidae)

**DOI:** 10.1080/23802359.2017.1280701

**Published:** 2017-02-16

**Authors:** David Jebb, Nicole M. Foley, Gerald Kerth, Emma C. Teeling

**Affiliations:** a School of Biology and Environmental Science, University College Dublin, Dublin, Ireland;; b Applied Zoology and Conservation, Zoological Institute, Greifswald University, Greifswald, Germany

**Keywords:** Chiroptera, myotis, mitogenome

## Abstract

In this study, we present the complete mitochondrial genome of the Bechstein’s bat, *Myotis bechsteinii*. The mitogenome is 17,151 bp in length and is AT-rich with base composition A (27.8%), C (22%), G (16.1%), and T (34.1%). The mitogenome shows conserved gene content and order similar with other mammalian mitogenomes, being composed of 13 protein-coding genes, 2 ribosomal RNA genes, 22 transfer RNA genes, and one control region. The majority of genes are encoded on the H-Strand except for *ND6* and 8 tRNAs as found in other bat species. The field identification of *Myotis bechsteinii* was confirmed by phylogenetic analyses using datasets comprising whole mitogenomes and *COXI*. This mitogenome is a resource for future studies of *Myotis* bats and other mammals.

*Myotis bechsteinii* is a small, insectivorous bat species, typically found in forest areas. *M. bechsteinii* were wild caught in a forest near the city of Würzburg, Germany (latitude: N49°43′, longitude: E9°49′), where the species has been studied for more than two decades (Kerth & van Schaik 2012). A 3 mm diameter wing biopsy was taken and stored in silica beads prior to DNA extraction. DNA was extracted from an individual sample, DE6 (Teeling DNA Repository, UCD) using the Promega Wizard SV96 Genomic DNA purification system (catalogue no. A2371, Promega Corporation, Madison, WI). The entire mitogenome was amplified and sequenced using the primers, protocols, and sequencing procedures previously reported in Jebb et al. (2015).

The entire mitogenome (GenBank accession no. KX757757) was assembled *de novo* using ABySS (v 1.5.2) (Simpson et al. 2009). The mitogenome was annotated using ARWEN (Laslett & Canbäck 2008) and Geneious (v 7.1.7) (Kearse et al. 2012), with *M. myotis* (Jebb et al. 2015) as a reference.

Following recommendations in Botero-Castro et al. (2014), we phylogenetically validated the *M. bechsteinii* mitogenome. First, all *Myotis COXI* barcode sequences were downloaded from the Barcode Of Life Database (BOLD), and the assembled *M. bechsteinii COXI* sequence was added with a *Murina ussuriensis COXI* providing an outgroup. After quality control, 360 sequences were aligned with MAFFT (Katoh & Standley 2013) and resulted in a final alignment of 649 bp. Second, the *M. bechsteinii* whole mitogenome was aligned to 56 published, bat mitogenomes, and one outgroup (cow) using MAFFT, and the alignment was filtered using Gblocks (Castresana 2000). For both datasets, the GTR + Γ+I model of substitution was chosen under the AIC in jModeltest2 (Darriba et al. 2012). RAxML (v7.2.8) (Stamatakis 2006) was used to construct Maximum Likelihood (ML) phylogenies with 1000 bootstrap replicates to estimate support. The ML whole mitogenome phylogeny is shown in Figure 1. MrBayes (v 3.2.6) (Huelsenbeck & Ronquist 2001) was used to construct a whole mitogenome phylogeny using Bayesian Inference (BI). Two iterations with 4 chains were run for 1.1 million generations and sampled every 200, with the first 100,000 generations discarded as burn-in. After 1.1 million generations, the analysis had reached stationarity and the iterations converged.

**Figure 1.**
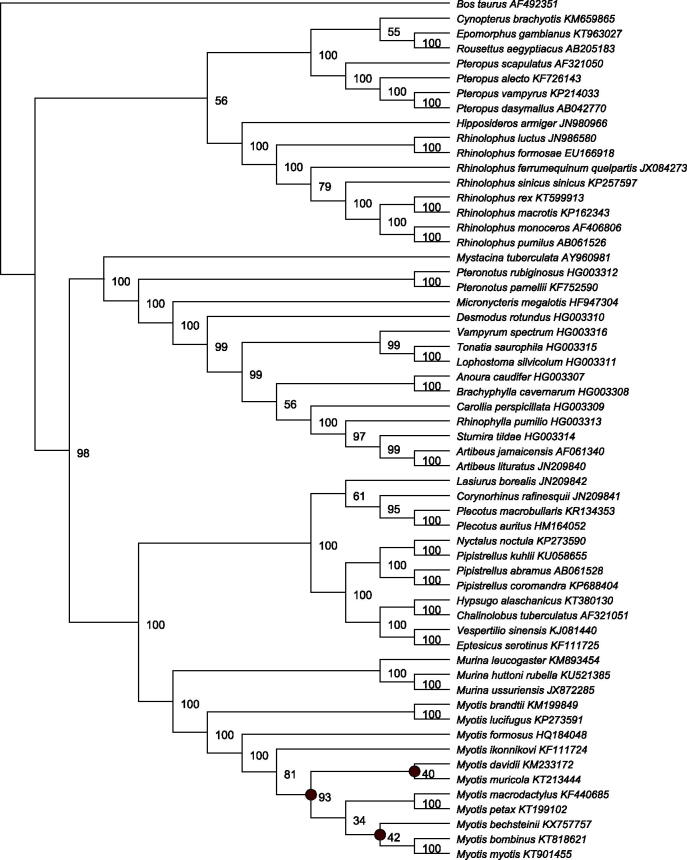
Whole mitogenome, Chiroptera phylogeny constructed using RAxML from an alignment of 57 Chiroptera mitogenomes, with cow (*Bos taurus*) as an outgroup. GenBank accession numbers are shown adjacent to species names. Percentage bootstrap support is shown at each node. Both ML and BI analyses show *M. bechsteinii* clusters within the genus *Myotis*. Nodes within *Myotis* which differed between the two analyses are marked with grey circles.

The assembled *M. bechsteinii COXI* sequence grouped exclusively with *M. bechsteinii* barcodes in the *COXI* only dataset, confirming species identity. In the whole mitogenome phylogeny, the *M. bechsteinii* mitogenome was placed within the *Myotis* genus, as shown in Figure 1. The deep divergences within *Myotis,* amongst the Old World Clades, were poorly supported in both ML and BI analyses, and the exact relationships between the Eurasian clades differed between methods. These poorly supported deep nodes were previously reported by Ruedi et al. (2013), and are unlikely to be resolved with a purely mitochondrial dataset such as this.

In this study, we sequenced, assembled, and phylogenetically validated the mitogenome of *M. bechsteinii*, a species whose population genetic structure and social behaviour has been intensively studied (Kerth & van Schaik 2012). This mitogenome is a resource for phylogenetic, biomedical, and on-going conservation studies.
